# Ocular melanoma and mammary mucinous carcinoma in an African lion

**DOI:** 10.1186/1746-6148-8-176

**Published:** 2012-09-25

**Authors:** Didier Q Cagnini, Breno S Salgado, Juliana L Linardi, Fabrizio Grandi, Rafael M Rocha, Noeme S Rocha, Carlos R Teixeira, Fabio Del Piero, Julio L Sequeira

**Affiliations:** 1 Departamento de Clínica Veterinária, Faculdade de Medicina Veterinária e Zootecnia, University Estadual Paulista – UNESP, Botucatu, Brazil; 2 Departamento de Patologia, Faculdade de Medicina de Botucatu, University Estadual Paulista – UNESP, Botucatu, Brazil; 3 Curso de Medicina Veterinária, Fundação de Ensino e Pesquisa de Itajubá – FEPI, Itajubá, Brazil; 4 Department of Diagnostic Pathology, Public Veterinary Hospital, Veterinary Service of National Association of Small Animal Clinicians (Anclivepa), São Paulo, SP, Brazil; 5 Departamento de Anatomia Patológica, Hospital A.C. Camargo. Fundação Antônio Prudente, São Paulo, Brazil; 6 Department of Pathobiological Sciences, Louisiana State University, School of Veterinary Medicine, Baton Rouge, LA, USA

**Keywords:** *Panthera leo*, Ocular neoplasms, Mammary gland, Melanocytic neoplasms, Peribiliary cysts

## Abstract

**Background:**

Reports of neoplasms in *Panthera* species are increasing, but they are still an uncommon cause of disease and death in captive wild felids. The presence of two or more primary tumor in large felids is rarely reported, and there are no documented cases of ocular melanoma and mammary mucinous carcinoma in African lions.

**Case presentation:**

An ocular melanoma and a mammary mucinous carcinoma are described in an African lion (*Panthera leo*). The first tumour was histologically characterized by the presence of epithelioid and fusiform melanocytes, while the latter was composed of mucus-producing cells with an epithelial phenotype that contained periodic acid-Schiff (PAS) and Alcian blue staining mucins. Metastases of both tumor were identified in various organs and indirect immunohistochemistry was used to characterize them. Peribiliary cysts were observed in the liver.

**Conclusions:**

This is the first description of these tumor in African lions.

## Background

Reports of neoplasms in *Panthera* species are increasing, particularly due to the increase in longevity of captive animals as well as the increased use of ultrasound, routine physical examinations, complete blood counts, blood chemistries, diagnostic biopsies and necropsies. Various neoplasms have been reported in captive lions. These include cutaneous tumor such as basal cell epithelioma [1], mast cell tumor [2], and subcutaneous fibrosarcoma [3]. There are also reports of other neoplasms such as oligodendroglioma [4], mucoepidermoid carcinoma of the salivary gland [5], gastric carcinoma [6], mammary carcinoma with ductular differentiation [7], lymphoma [8], intestinal adenocarcinoma with peritoneal carcinomatosis [9], and gall bladder carcinomas [10]. Recently, peribiliary cysts were also recognized in African lions [11,12].

The presence of two or more primary tumor in wild felids has rarely been reported [13,14] and there are no documented cases of ocular melanoma and mammary mucinous carcinoma in African lions. This report describes the clinical, histological, and immunohistochemical features of an ocular melanoma of the anterior chamber and of a spontaneous mammary mucinous carcinoma in an African lion (*Panthera leo*).

## Case presentation

A 19-year-old captive ovariohysterectomized female African lion from a zoological park in Brazil was presented at the Veterinary Hospital of the São Paulo State University, Botucatu, Brazil, with the suspicion of an intraocular neoplasm with liver metastasis. Conjunctival hyperemia, moderate serous epiphora, buphthalmia and presence of an intraocular mass were detected in the left eye. Additionally, the lioness had a history of progressive weight loss, regurgitation, vomiting, and chronic fatigue. Biochemical analysis revealed an increase in alkaline phosphatase [145.8 IU/L; Reference: 0–96 IU/L], gamma glutamyltransferase [42.4 IU/L; Reference: 0–17 IU/L], and alanine aminotransferase [246.2 IU/L; Reference: 0–195 IU/L]. Reference values were based on the ISIS species value references [15]. Hematologic parameters (PCV, RBC, WBC, hemoglobin, mean cell hemoglobin concentration and serum total protein) were within normal limits.

Ocular enucleation was performed and the ocular globe was submitted for histological evaluation to the Veterinary Pathology Service of the same institution. Grossly, a firm, mottled black to white mass, measuring 1.5 cm in diameter infiltrated the anterior uvea filling approximately 30% of the anterior chamber. Tissue samples were collected, fixed in 10% neutral buffered formalin and sent for histopathological examination. Four-μm-thick sections were stained with haematoxylin and eosin and were histologically evaluated. The mass was composed of solid sheets of oval to epithelioid cells supported by scant fibrovascular stroma. The cells had moderate amounts of cytoplasm and indistinct cell borders. There was scattered intracytoplasmic melanin pigmentation that sometimes obliterated the cellular features. The majority of the cells presented oval nuclei with coarse stippled chromatin and an irregular and single nucleolus. Moderate anisocytosis and anisokaryosis were seen and there were approximately 3 mitotic figures per high power field. No atypical mitoses were observed. The morphologic features examined lead to a diagnosis of intraocular melanoma.

An abdominal ultrasound examination revealed multiple anechoic areas surrounded by thin hyperechoic structures interpreted as capsules surrounding cavitary areas. Hypoechoic nodules were also observed. Liver samples were then obtained by ultrasound-guided fine-needle aspiration for cytopathologic evaluation. Direct smears were prepared and stained with Giemsa (Merck, Darmstadt, Germany). Cytologically, there were hepatocytes containing intracytoplasmic lipid droplets indicating hepatic lipidosis, which was expected given the age of the lioness. During the liver fine-needle aspiration biopsy, a subcutaneous enlargement in the region of the right inguinal mammary gland measuring 7 × 3 cm was noted. Samples for cytopathologic evaluation were obtained by fine-needle aspiration of the mass and the cell morphology was suggestive of a mammary carcinoma.

Given the deterioration of the lioness’ clinical condition characterized by cachexia, the animal was euthanatized 2 months after the initial diagnosis of the mammary neoplasm, and was presented at the Veterinary Pathology Service of the same institution for post-mortem examination. At necropsy, an encapsulated subcutaneous lobular mass in the mammary region, measuring 25 × 13 × 5 cm was observed. On cut surface, the tumor was moderately firm, mottled white to yellow, and contained multifocal cysts measuring from 0.4 to 2 cm in diameter. Inguinal lymph nodes were enlarged measuring 2 × 9 × 3 cm, and presented multifocal lesions similar to the mammary nodule on cut surface. No additional cutaneous or subcutaneous masses were observed.

**Figure 1:**
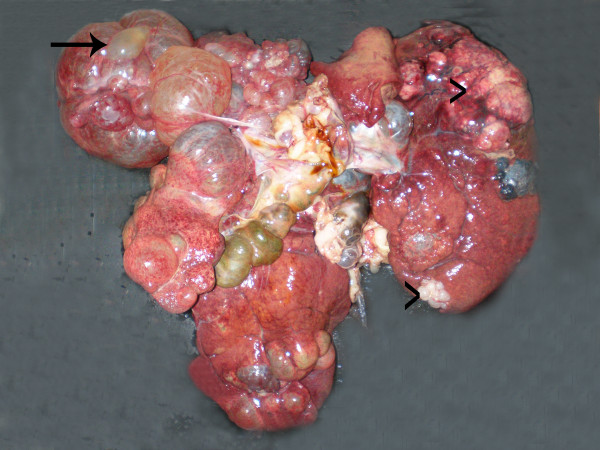
Cystic (arrow) and solid formations (arrowheads) can be observed in the liver.

The liver had multifocal to coalescing cystic areas and masses (Figure 1). The cysts measured from 0.5 to 12 cm in diameter and contained a translucent fluid. Masses were moderately firm, white to yellow, measured from 2 to 12 cm in diameter, and had multifocal petechiae and ecchymoses on cut surface.

Tumor similar to the mammary and liver masses were observed in the lungs and pleura and measured 1–4 cm. No other abnormalities were observed.

**Figure 2:**
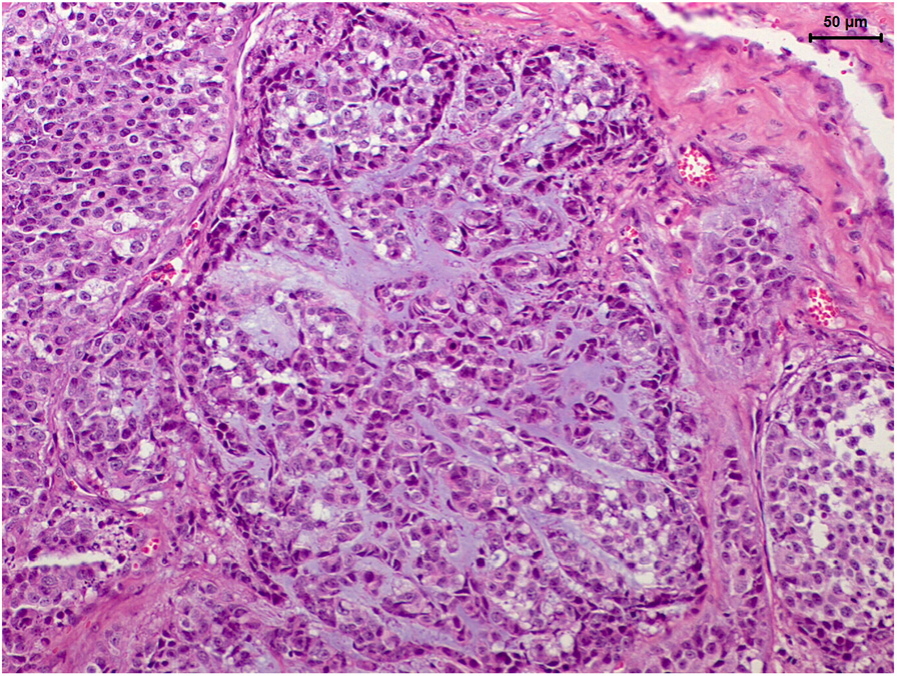
**Basophilic to amphophilic extracellular mucus and slightly eosinophilic intracellular mucus can be observed in the mammary neoplasm.** Hematoxylin and eosin (bar = 50 μm).

Samples were collected and processed for histologic examination. Microscopically, the mammary neoplasm was composed of clusters of cuboidal epithelial cells surrounded by a robust fibrovascular stroma separating the tissue in lobules. The cells presented moderate amounts of cytoplasm with indistinct cell borders, oval nuclei with a coarse stippled chromatin and multiple prominent nucleoli (Figure 2). Occasional cells with signet-ring appearance could be observed. There were small lakes of basophilic to eosinophilic proteinaceous material and multifocal necrosis of neoplastic tissue (Figure 2).

The histochemical features of the fluid were assessed by PAS, PAS-diastase, and alcian blue pH 2.5 stains. Intestinal and bronchial mucosal tissues from domestic cat and lion were used as positive controls for mucin. In the present case, PAS, with and without diastase, and alcian blue pH 2.5 positive mucins were observed within cells and stroma. Based on gross and histologic examination, the tumor was considered to be a spontaneous mammary mucinous carcinoma.

The liver contained solid neoplastic areas composed of medium size round to larger epithelioid oval cells with abundant cytoplasm, vesicular nuclei, and single prominent nucleoli supported by a fibrovascular stroma. Intracytoplasmic melanin was detected in a few neoplastic cells. Sections of the cystic areas had single layers of epithelial cells surrounded by fibrous connective tissue and were primarily located around the portal areas and were interpreted as peribiliary cysts. Lungs and pleura contained metastatic foci histologically similar to the primary mammary and ocular neoplasms.

To characterize the primary neoplasms and the metastatic foci, 3 μm serial sections were obtained and used for indirect immunohistochemistry. Antigen retrieval was performed by heat treatment in 10 mM citrate buffer, pH 6.0 for all primary antibodies. A polymeric labeling system kit (NovoLink Polymer Detection System, Novocastra Laboratories, Newcastle, UK) was used for peroxidase and protein blockages and detection of antigen-antibody reactions in all slides. Slides were incubated overnight at 4°C with specific primary antibodies. The primary antibodies used consisted of monoclonal antibodies against cytokeratin (Dako, clone AE1/AE3, diluted 1: 500), cytokeratin 19 (LabVision, clone BA17, diluted 1:400), α-smooth muscle actin (Santa Cruz Biotechnologies, clone 1A4, diluted 1:1200), vimentin (Dako, clone V9, diluted 1:2000), and melan A (Dako, clone M7196, diluted 1:50). Sections of normal domestic cat, lion mammary gland were used as positive controls for all antibodies except melan A, for which a canine cutaneous melanoma and domestic cat, lion pigmented skin were used. For negative control purposes the primary antibody was replaced by non-immune rabbit IgG (Dako). The slides were immersed with the detection system following the manufacturer’s instructions and 3,3’ diaminobenzidine tetrahydrochloride was used as chromogen in order to allow the visualization of antigen-antibody reaction. The slides were then counterstained using Harris’s hematoxylin, dehydrated, and mounted for evaluation via light microscopy.

The intraocular neoplastic cells expressed vimentin and Melan A, while cytokeratin, cytokeratin 19, and α-smooth muscle actin were not expressed. This immunophenotype indicated melanoma. The mucin-producing cells of the mammary tumor had an epithelial phenotype with cytokeratin 19 expression in the membrane and cytoplasm. These cells were also strongly vimentin positive (including mucin-producing cells) and mildly α-smooth muscle actin positive. Nonmucin-producing mammary cells were also detected and expressed cytokeratin 19, vimentin and α-smooth muscle actin. Stromal cells expressed vimentin and were negative for cytokeratin 19 and α-smooth muscle actin and were interpreted as fibroblasts. Given the immunohistochemical results, the mammary tumor was characterized mucinous carcinoma.

**Figure 3:**
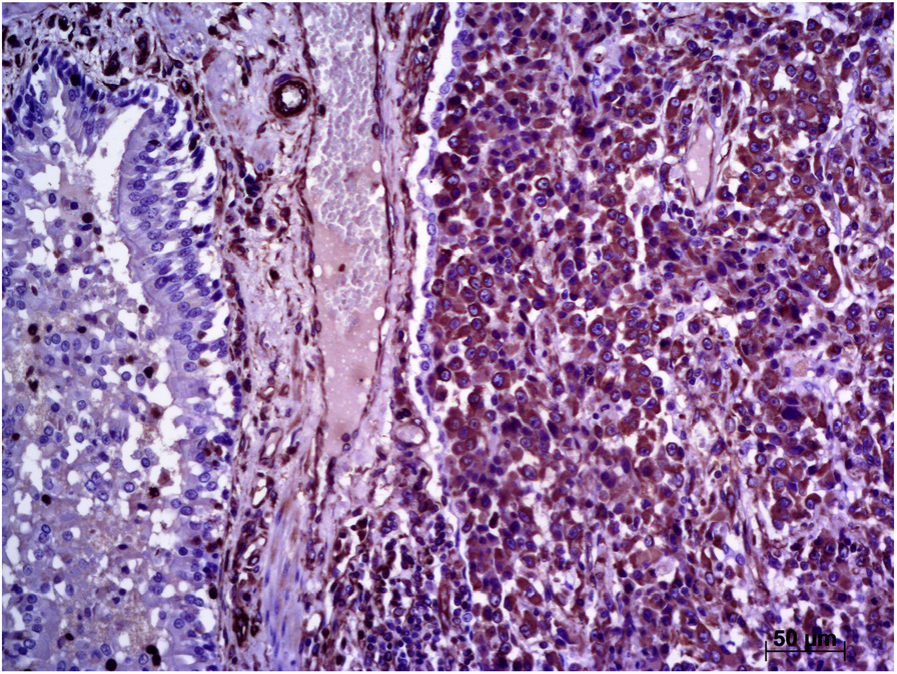
**Melanoma cells expressing vimentin obliterating the liver architecture.** DAB indirect immunohistochemistry, Harris hematoxylin counterstain (bar = 50 μm).

Metastatic foci in the lungs were also assessed using immunohistochemistry. Epithelial cuboidal cell clusters immersed in a mucinous stroma expressed cytokeratin, cytokeratin 19 and vimentin, an immunohistochemical profile consistent with mammary cells. In contrast, areas with epithelioid single cells expressed vimentin and Melan A, indicating melanoma cells. This indicated the presence of both mammary and melanoma pulmonary metastases. Multifocal melanoma liver metastases were identified by lack of expression of cytokeratin, cytokeratin 19 and the expression of Melan A and vimentin (Figure 3).

The presence of two or more primary tumor in wild felids is rarely reported. Multiple myeloma, adrenocortical carcinoma, and pheochromocytoma in a Jaguar (*Panthera onca*) [13], seminoma and parathyroid adenoma in a Snow leopard (*Panthera uncia*) [14], and mammary carcinoma and leiomyosarcoma in a Jaguar [16] were previously documented. There are no reports of multiple neoplasms in African lions (*Panthera leo*). Additionally, there are no documented cases of simultaneous and isolated occurrence of ocular melanoma and mammary mucinous carcinoma in these animals.

Tumor of melanocytic origin are the most common primary intraocular neoplasms of cats [17]. Ocular melanomas are tumor that tend to occur in the anterior uveal tract of felines, originating within the iris [18-20], which was the possible origin in this case since it was affecting the anterior ocular chamber. In large felids, there are no data regarding ocular melanomas. Ocular melanomas can be locally infiltrative and may metastasize widely in cats [19,21]. Data concerning metastatic potential and rate of metastasis in feline ocular melanomas are controversial. Although, some authors described that ocular melanomas in cats posses a high metastatic behavior [22,23] attention should be paid to the fact that there are few long term survival studies regarding rate of metastasis in this type of cancer. In an observational study in domestic cats with diffuse iris melanomas, authors were not able to confirm with a satisfactory degree of reliability if the causes of death were truly associated with metastatic disease [24]. In this case, metastases to the liver and lungs were observed as has been described in cats. Neoplastic cells of the ocular neoplasm expressed Melan A and vimentin, similarly to those observed in domestic felines’ melanomas [25].

Mammary carcinomas were described in wild felids [26,27] and were more common in animals that had been implanted with melengestrol acetate (MGA)-impregnated silastic devices [26]. Mammary mucinous carcinomas are tumor of the mammary gland characterized by presence of abundant mucin. This histologic pattern was described in a Tiger [28] and is rarely reported in the veterinary literature [29-31]. Their prominent feature is the presence of large amounts of mucinous material with tintorial affinities to periodic acid-schiff (PAS), with and without diastase, and alcian blue stains [32-36]. It was asserted that the predominant feature of this neoplasm is the presence of large amounts of mucinous material that stains positive to PAS, with and without diastase, and alcian blue stains [36]. In this case, positive staining of the mucus with PAS-diastase and an epitelial cytokeratin staining pattern indicated the presence of a mammary mucinous carcinoma.

Mucin-producing cells expressed cytokeratin, cytokeratin 19 and vimentin. This is similar to those observed in humans [37] and domestic felines, where luminal vimentin-positive epitelial cells were described [30].

This event is a consequence of neoplastic progression participating in the metastatic cascade and indicating a poor prognosis [38-40]. Additionally, vimentin expression in human breast carcinomas is considered a malignancy indicator [37]. It is well known that feline mammary tumor express vimentin and that most of them are malignant [30]. Consequently, similar features apply to other felids’ mammary neoplasms.

Peribiliary cysts were observed in the liver. The cysts were primarily located in portal areas, with abundant fibrous tissue, and a single layer of epithelial cells. Differentiating biliary cysts from biliary cystadenoma can be challenging since the two entities are rather similar grossly and histologically [11]. Biliary cystadenoma is located within the parenchyma, whereas peribiliary cysts are located in the connective tissue of the hepatic hilus and also within the large intrahepatic portal tracts. Also, the typical biliary cystadenoma of domestic animals has a smaller amount of stromal content, in contrast to this case that exhibited abundant stromal component around the cysts. In humans, peribiliary cysts can have a genetic predisposition, or are associated with hepatobiliary disease, and exposure to chemical carcinogens [41,42]. Peribiliary cysts have already been reported in African lions [11,12] and there are no data regarding their aetiology in animals. Perhaps they are predisposed by the melanoma metastases in the liver in this case. Metastatic nodules could obliterate the necks of the peribiliary glands, resulting in the formation of retention cysts, as described by others [41].

The most plausible explanations for two different tumor in the same or separate organs are clonal variants of the same neoplasm, metastatic populations from separate tissue types of a mixed tumor, or metastases of two completely separate tumor. In this lioness, the tumor in her liver and lungs were grossly similar and required histological and immunohistochemical examination for better characterization. Immunohistochemistry is a useful tool to confirm the cell origin and differentiate multiple neoplasms and metastasis. In the present case, Melan A indirect immunohistochemistry was able to correctly differentiate between metastatic melanoma and mammary cell populations.

## Conclusions

This is the first case in an African lion where there were two simultaneous neoplasms had metastasized. Additionally, this is the first case in which peribiliary cysts were associated with obliteration of hepatic tissue due to a metastatic neoplasm. Immunohistochemistry is a good ancillary tool for differentiation of cell populations in metastatic foci.

## Competing interests

The authors declare that they have no competing interests.

## Authors’ contribution

DQC, BSS, FG, NSR, FDP and JLS supervised the anatomy and pathological evaluation, reviewed the literature, and prepared the manuscript. JLL and CRT carried out the clinical examination. DQC, BSS, and RMR performed the immunohistochemical staining. All authors read and approved the final manuscript.
